# Genome-Wide Screen of Three Herpesviruses for Protein Subcellular Localization and Alteration of PML Nuclear Bodies

**DOI:** 10.1371/journal.ppat.1000100

**Published:** 2008-07-11

**Authors:** Jayme Salsman, Nicole Zimmerman, Tricia Chen, Megan Domagala, Lori Frappier

**Affiliations:** 1 Department of Molecular Genetics, University of Toronto, Toronto, Ontario, Canada; 2 Affinium Pharmaceuticals Inc., Toronto, Ontario, Canada; Oregon Health & Science University, United States of America

## Abstract

Herpesviruses are large, ubiquitous DNA viruses with complex host interactions, yet many of the proteins encoded by these viruses have not been functionally characterized. As a first step in functional characterization, we determined the subcellular localization of 234 epitope-tagged proteins from herpes simplex virus, cytomegalovirus, and Epstein–Barr virus. Twenty-four of the 93 proteins with nuclear localization formed subnuclear structures. Twelve of these localized to the nucleolus, and five at least partially localized with promyelocytic leukemia (PML) bodies, which are known to suppress viral lytic infection. In addition, two proteins disrupted Cajal bodies, and 19 of the nuclear proteins significantly decreased the number of PML bodies per cell, including six that were shown to be SUMO-modified. These results have provided the first functional insights into over 120 previously unstudied proteins and suggest that herpesviruses employ multiple strategies for manipulating nuclear bodies that control key cellular processes.

## Author Summary

Herpes simplex virus, Epstein–Barr virus, and cytomegalovirus are three types of human herpesviruses that infect most people for their entire life and, under some circumstances, cause significant diseases. Each virus encodes a large number of proteins that function to manipulate the host cell to the best advantage of the virus; however, many of these encoded proteins have never been studied. We have generated constructs to express most of the proteins encoded by these three viruses in human cells and have determined the precise localization of each in the cell. We have also examined how each viral protein affects host nuclear structures called PML bodies, which are part of the cellular response to suppress viral replication. We identified several proteins from all three viruses that disrupt PML bodies, suggesting that they would enable viral infection. Our study has given the first information on the potential function of 120 previously unstudied viral proteins and shows that each virus has multiple mechanisms to disrupt PML bodies that were not previously recognized.

## Introduction

Herpesviruses are large DNA viruses that each encode between 80 and >200 proteins. This complex network of proteins results in a variety of effects on the host cell and ensures efficient proliferation of the virus. While the functions of a subset of these proteins are reasonably well understood, there is little or nothing known about the function of many of the proteins. Understanding the role that each viral protein plays is not only useful for determining how the virus propagates and potentially inhibiting that propagation, but also for identifying mechanisms that regulate key cellular processes.

Herpesviruses are divided into alpha, beta and gamma subfamilies, which differ considerably in their modes of latent infections and in their effects on the host cells. These studies involve one member of each of the three subfamilies of herpesviruses; herpes simplex type 1 (HSV-1) of the alphaherpesviruses, human cytomegalovirus (CMV) of the betaherpesviruses and Epstein-Barr virus (EBV) of the gammaherpesviruses. All three of these viruses can cause significant human disease in addition to providing interesting model systems for viral-host interactions. HSV-1 and EBV encode 80–85 proteins, while the larger CMV encodes approximately 200–230 proteins (depending on the strain). Beyond a set of core genes found in all herpesviruses, the remaining genes are specific to either the subfamily or individual virus and likely contribute to their individual and complex replication strategies. The high number of CMV proteins relative to those of HSV and EBV clearly reflects aspects of CMV infection that must be unique to this virus or subfamily, and many of these proteins have no assigned function; in fact, over 60% of the CMV-encoded proteins have no functional annotation. A better understanding of the individual protein functions would be useful for a more mechanistic understanding of the commonalities and differences in these three viruses.

Determination of subcellular localization is one way that the potential roles of large numbers of proteins can be assessed [1]. Such global screening of subcellular localization has been successfully conducted with yeast and human proteins, where individual proteins were expressed fused to epitope or to GFP tags [2]–[4]. For most proteins, localization results were found to be consistent in different studies regardless of the tag used or expression level. Subcellular localization screening is also an appropriate starting point for the characterization of herpesvirus proteins, providing insight into the potential contribution of the protein to viral infection as well as the cellular processes that may be manipulated. Although the localization is known for the majority of HSV proteins, the localization is known for only about 50% of the EBV proteins and 10% of CMV proteins, leaving over 200 proteins from these 3 viruses that are uncharacterized.

Successful herpesvirus infections require manipulation of the host cell processes to favor viral infection, including alterations of host nuclear bodies (NBs). Indeed many RNA and DNA viruses are known to target various host NBs as part of their replication strategies. For example, adenoviruses and herpesviruses encode several proteins that cause redistribution of nucleolar components, reorganization of the nucleolus and interference with nucleolar function, including disrupting rRNA synthesis, processing and trafficking [5],[6]. Cajal bodies, which are important for the maturation of nuclear RNP complexes, are site of localization of the avian herpesvirus MDV Meq protein [7] and are reorganized by adenovirus infection [8],[9] and by groundnut rosette virus ORF3 [10]. A few herpesvirus proteins have been reported to associate with nuclear speckles (also called SC35 domains or splicing speckles), nuclear structures that are storage sites for splicing factors and, in some cases, alter nuclear speckles as a reflection of their effects on cellular transcription and splicing [11]–[16].

Perhaps the most notable NB affected by viral infection are promyelocytic leukemia (PML) bodies (also called ND10s or PODs). PML NBs are involved in several important host cell processes including apoptosis and the DNA damage response [17]. In addition, they suppress lytic infection of both RNA and DNA viruses as part of an intrinsic immune response and may also regulate quiescent viral infections [18]–[22]. For example, overexpression of a PML isoform has been shown to inhibit the replication of Human Foamy Virus, Vesicular Stomatitis Virus (VSV) and influenza virus, while silencing of PML enhances the propagation of influenza virus and CMV [18],[21],[22]. Also PML-deficient mice are more susceptible to VSV, rabies virus and lymphocytic choriomeningitis virus infections [23]–[25]. To counteract this antiviral response, some viral proteins disrupt PML NBs, often through degradation or dispersal of the PML protein that forms the core of PML NBs [15],[16],[26]. For herpesviruses, HSV ICP0, CMV IE1 and EBV BZLF1 proteins have been found to disrupt PML bodies through effects on the PML protein, and this effect of ICP0 and IE1 has been shown to correlate with increased lytic viral expression and replication [22], [27]–[31]. While a small number of herpesvirus proteins have been identified that contribute to viral infection through alteration of host NBs, many proteins have never been examined for these effects.

To gain a more comprehensive understanding of the many functions of herpesvirus proteins, we have generated a mammalian expression library consisting of most of the open reading frames of HSV, CMV and EBV. These proteins are expressed fused to a C-terminal tag suitable for protein localization and protein interaction studies. We present the subcellular localization screening for all of these proteins. We also identify viral proteins that localize to and/or alter host NBs, including several that had not previously been characterized.

## Results

### Expression libraries

In order to conduct genome-wide studies on herpesvirus proteins, we attempted to clone each of the predicted ORFs from HSV-1, CMV (strain AD169) and EBV into a mammalian expression vector from which proteins are expressed fused to a C-terminal sequential purification affinity (SPA) tag comprised of a calmodulin binding peptide and a triple FLAG epitope [32]. Such constructs are suitable for both protein localization and protein interactions studies. In addition, nineteen CMV ORFs (UL133-151) not present in the AD169 laboratory strain were cloned from the Towne strain of CMV. While not every potential ORF was recovered in the high throughput cloning effort, we generated expression constructs for the majority of each genome namely, 60 HSV, 61 EBV, 148 CMV ORFs. A comprehensive list of the individual proteins used in this study is provided as Table S1. We examined the migration of approximately half of the viral proteins in Western blots to verify the quality of the expression library and found that 91% (103 out of 113) migrated at the predicted size or larger (see sample blots in Figure S1), with the remaining 9% ran faster than expected (perhaps due to protein truncation, proteolysis or anomalous migration). Note that anomalously slow migration in SDS-PAGE is a common property of highly charged proteins and is consistent with the highly basic or acidic nature of most of the viral proteins that we found to migrate slower than their predict molecular weight.

### Subcellular localization of herpesvirus proteins

**Figure 1:**
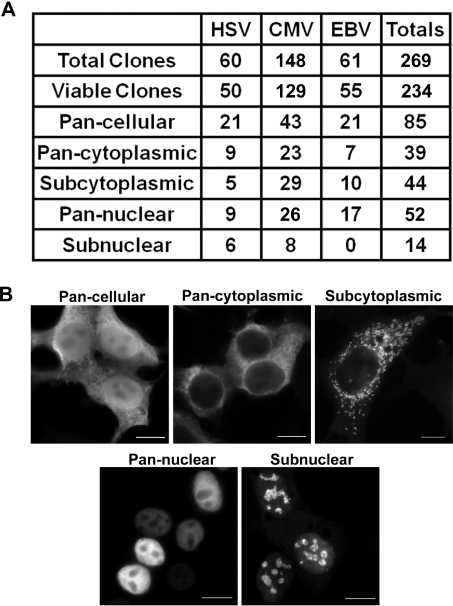
Localization summary of herpesvirus proteins expressed in 293T cells. (A) Summary table indicating the localizations of herpesvirus proteins expressed in transfected 293T cells as determined by IF detection of the C-terminal FLAG epitope. Viable clones refers to the number of clones for which the viral protein was detected by IF. (B) IF micrographs representative of the pan-cellular, pan-cytoplasmic, subcytoplasmic, pan-nuclear and subnuclear localizations. Scale bar  =  10 µm.

We attempted to determine the subcellular localization for all of the viral proteins expressed in 293T cells, by transient transfection of the expression plasmids and immunofluorescence (IF) microscopy for the FLAG epitope (Figure 1A). We obtained localization data for 234 proteins from the 269 expression plasmids tested, of which 148 (14 HSV, 100 CMV, 34 EBV) have not been previously characterized in terms of their localization and most of these also lack any functional characterization. The localization of 35 of the viral proteins could not be determined due to protein expression below detectable levels and/or cell toxicity. Of the 234 viral proteins we visualized, 85 have previously published subcellular localization data, and there is excellent agreement between our results and previous reports (see Table S1 for individual protein results). Minor discrepancies in localization were observed for about 15 proteins with only 2 proteins (1.3%; HSV UL56 and EBV BRRF1) being exclusively in subcellular compartments different from previous reports. To determine the likelihood of the protein localization being altered due to our C-terminal tag, we also compared our localizations to those in the literature for individual proteins expressed without a tag. Twelve such proteins (4 HSV and 8 CMV) were identified and all had localizations consistent with ours (see Table S1). Some discrepancies would be expected with localizations determined in the context of viral infection since interactions between viral proteins can alter the localization of the individual proteins. Therefore we also compared our localization data to that determined for untagged viral proteins in the context of viral infection. 24 of the HSV, 12 CMV and 9 EBV proteins in our study had been previously localized in the context of an infection using specific antibodies. Two of these proteins (HSV UL19 and UL56) had localizations that were inconsistent with our observations, while seven more (HSV ORFP, UL29, UL30, UL31, UL35, UL51, US12) had staining patterns that overlapped with but were more restricted than the patterns we observed (see Table S1). Differences in localization could be due to the presence of additional viral proteins during infection, differences in protein expression levels or the presence of our epitope tag. As expected, this initial analysis shows that such high through-put localization screening will not accurately determine the localization of every viral protein in the context of infection, however it does show that such an approach is appropriate for predicting the localization of the vast majority of viral proteins even in the context of infection.

Protein localizations were broadly categorized as either pan-cellular, pan-cytoplasmic, subcytoplasmic, pan-nuclear or subnuclear. Some proteins had more complex localization patterns and fell into multiple categories (e.g. HSV UL24 is subcytoplasmic and subnuclear) and, in these instances, the protein was recorded in the most prevalent category and the localization pattern detailed in the notes section of Table S1. The results for all proteins are summarized in Figure 1A and detailed in Table S1. Most of the viral proteins were diffusely localized throughout the nucleoplasm (52 proteins), throughout the cytoplasm (40 proteins) or throughout both compartments, often at unequal levels (87 proteins). In addition, 44 proteins localized to subcytoplasmic structures as shown in Figure S2. Five proteins (CMV UL12, UL59, US3, US5 and HSV UL12), appeared as vesicular-like bodies throughout the cytoplasm and seven proteins (HSV UL49, CMV UL77, UL135, US27 and EBV BcRF1, BORF2, BXLF1) formed foci or plaques. The remainder of the 44 subcytoplasmic proteins demonstrated staining patterns indicative of organelle association similar to that observed with the ER-localized protein disulphide isomerase (PDI), and many co-localized with this marker indicating association with the ER and/or secretory pathway (Table S1, Figure S3). Finally, 14 of the viral proteins screened in 293T cells localized to a variety of subnuclear structures with many adopting a nucleolar-like appearance (Figure 1).

There are 26 proteins that are considered to be conserved across all three families of herpesviruses [33], although in some cases the degree of homology is limited. We have localization data for all of the three homologues in HSV, CMV and EBV for six of these protein families (Table S2). Not unexpectedly, the homologues generally have the same subcellular localization with only two proteins (CMV UL103 and HSV UL31) showing staining that was slightly altered (but overlapping) with their homologues.

### Subnuclear localizations

**Figure 2:**
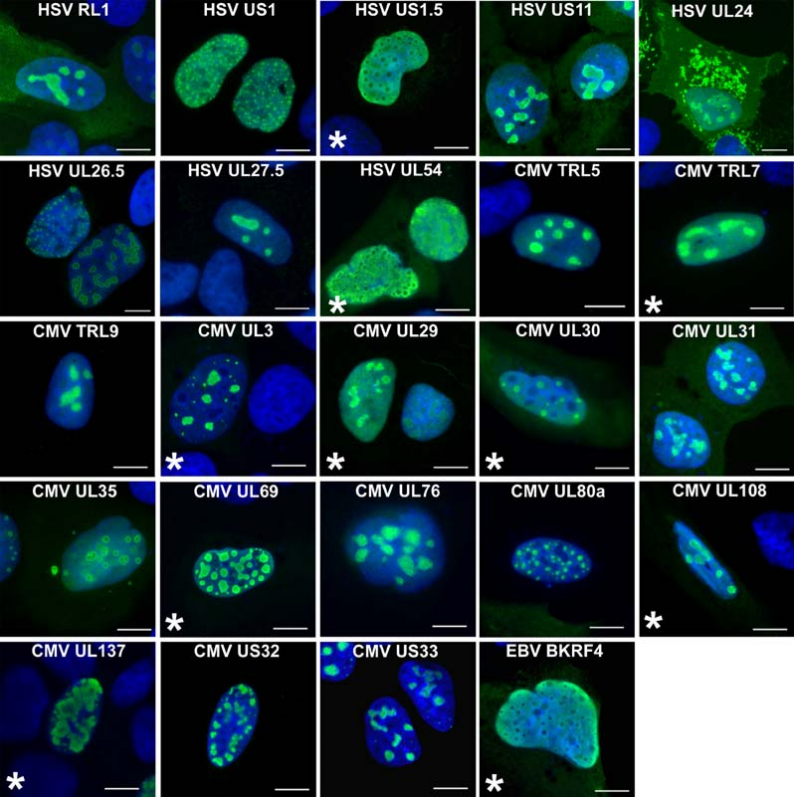
Herpesvirus proteins with subnuclear localization. U2OS cells transfected with expression plasmids for the indicated viral proteins (green) were fixed and stained with FLAG antibody to visualize the viral protein. DNA was visualized with DAPI stain (blue). For proteins marked with an asterisk (*), subnuclear structures were observed in U2OS cells but not in 293T cells while those not marked were observed in both U2OS and 293T cells. Scale bar  =  10 µm.

We are particularly interested in how herpesviruses manipulate host nuclear events and therefore further characterized the viral proteins that localized to the nucleus. Ninety-three proteins which were completely (66) or predominantly (27) nuclear in 293T cells, were reanalyzed in U2OS cells to verify their localization in another cell background, free of endogenous viral proteins. Results for individual proteins are indicated in Table S1. Eighty-three out of the 93 proteins had localizations in U2OS cells that were indistinguishable from those in 293T cells, including the 14 proteins found to be subnuclear in 293T cells (Figure 2, images without asterisk). The remaining 10 proteins, recorded as pan-nuclear or pan-cellular in 293T cells, also had the same localization in most U2OS cells, but in a fraction of the U2OS cells showed subnuclear localization (Figure 2, images with asterisks). We have added these to the list of sub-nuclear proteins, since they clearly have the capacity to form subnuclear structures under some circumstances, perhaps at higher expression levels. Two of the additional subnuclear proteins, HSV US1.5 and EBV BKRF4, did not actually form nuclear bodies but rather exhibited largely pan-nuclear staining with discreet foci that excluded the viral protein (see Figure 2 and Figure S4).

**Figure 3:**
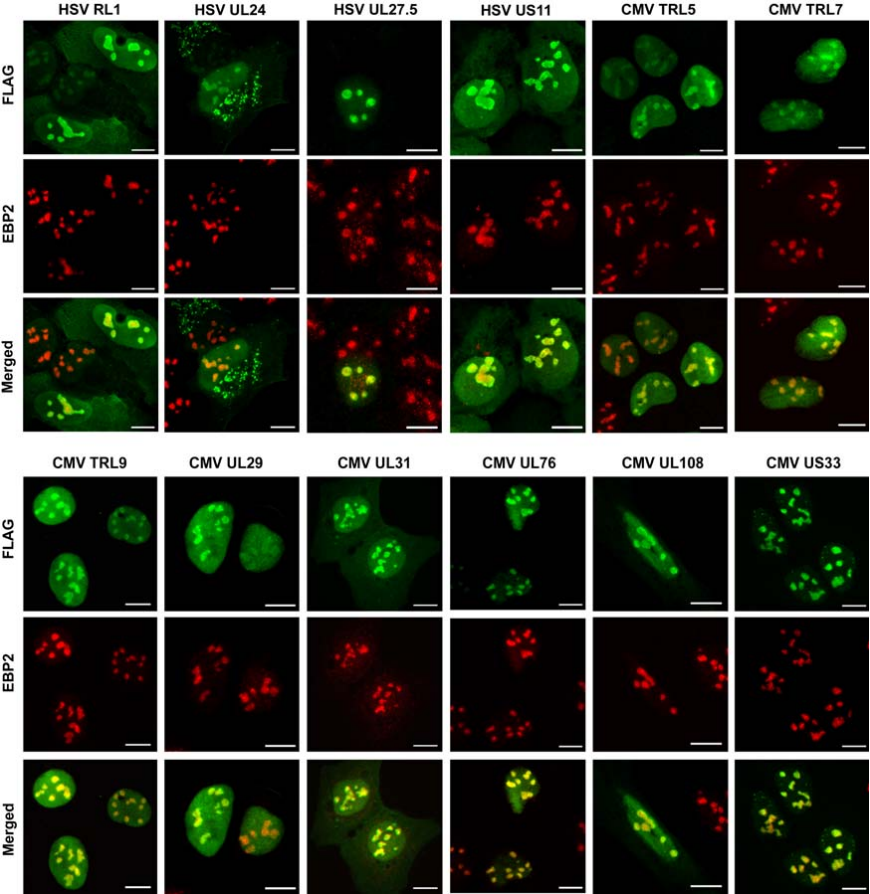
Herpesvirus proteins associated with the nucleolus. U2OS cells expressing the indicated viral proteins were stained with FLAG antibody to detect the viral protein (green) and with EBP2 antibody to visualize the nucleolus (red). Scale bar  =  10 µm.

In order to define the localization of the 24 subnuclear proteins more precisely and to determine how these proteins affect nuclear substructures, we analyzed the proteins by IF microscopy with markers for four nuclear bodies (nucleoli, PML NBs, nuclear speckles and Cajal bodies) that are targets of some viral proteins (Table 1). Several of the proteins had a nucleolar-like localization and hence were further examined for co-localization with EBP2 (EBNA-1 binding protein 2), a nucleolar protein involved in rRNA processing [34],[35]. Twelve of the 24 proteins tested co-localized with the EBP2 marker to varying degrees (Figure 3 and Table 1). Notably, CMV US33 was almost exclusively localized to the nucleolus, whereas all other proteins also demonstrated some nucleoplasmic localization. In addition, three HSV proteins, RL1 (ICP34.5), US11 and UL24 also showed significant cytoplasmic staining, including cytoplasmic structures in the cases of US11 and UL24.

**Table 1:** Subnuclear proteins and their association with host nuclear bodies.

	Nucleolus	Nuclear Speckles	PML Bodies	Cajal Bodies
**HSV RL1**	**Yes**	No	No	No
**HSV US1**	No	No	No	No
**HSV US1.5**	No	No	No	No
**HSV US11**	**Yes**	No	No	No
**HSV UL24**	**Yes**	No	No	No
**HSV UL26.5**	No	No	No	No
**HSV UL27.5**	**Yes**	No	No	No
**HSV UL54**	No	No	No	No
**CMV TLR5**	**Yes**	No	No	No
**CMV TLR7**	**Yes**	No	No	No
**CMV TLR9**	**Yes**	No	No	No
**CMV US32**	No	No	**Yes**	No
**CMV US33**	**Yes**	No	No	No
**CMV UL3**	No	No	**Yes**	No
**CMV UL29**	**Yes**	No	No	No
**CMV UL30**	No	No	No	No
**CMV UL31**	**Yes**	No	No	No
**CMV UL35**	No	No	**Yes**	No
**CMV UL69**	No	No	No	No
**CMV UL76**	**Yes**	No	No	No
**CMV UL80a**	No	No	**Yes**	No
**CMV UL108**	**Yes**	No	No	No
**CMV UL137**	No	No	No	No
**EBV BKRF4**	No	No	**Yes**	No

**Figure 4:**
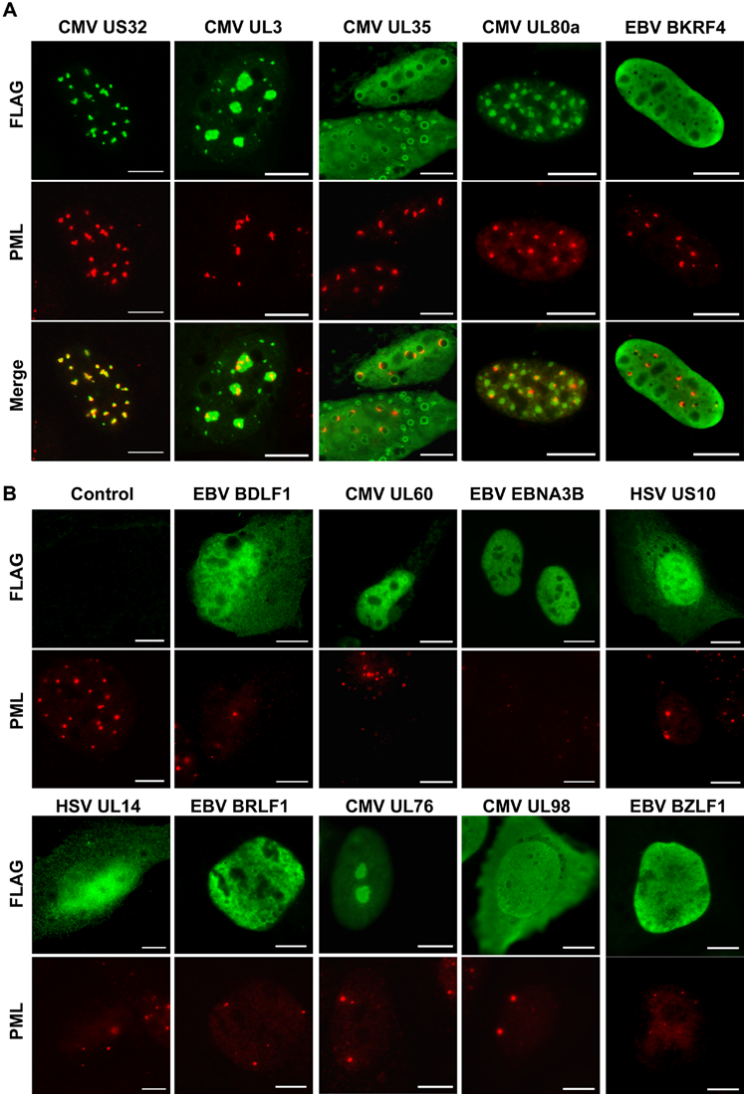
Effects of herpesvirus proteins on PML NBs. U2OS cells expressing the indicated viral proteins were stained with FLAG antibody to detect the viral protein (green) and with PML antibody to visualize the PML protein (red). (A) Viral proteins that exhibit some degree of co-localization with PML NBs. Images were also merged to show co-localization of viral proteins with PML bodies (yellow). (B) PML staining of untransfected control cells and viral proteins that disrupt PML NBs. Scale bar  =  10 µm.

We also examined subnuclear proteins for localization to PML NBs, Cajal NBs and nuclear speckles by co-staining for the main constituents of these bodies, namely PML, coilin and SC35 proteins, respectively. Five of the 24 subnuclear proteins co-localized at least partially with PML bodies (Table 1, Figure 4A). CMV US32 showed the most striking co-localization with PML, while CMV UL3 formed larger NBs that contained PML NBs and CMV UL80a formed foci that were often juxtaposed to PML NBs. CMV UL35 formed nuclear structures ranging from small round foci to larger spherical nuclear and cytoplasmic structures in which UL35 was excluded from the centre. PML co-localized with smaller UL35 foci and was found at the periphery of the larger spheres, following the contour of the structure. For EBV BKRF4, 10–20% of transfected cells exhibited diffuse nuclear staining with multiple small foci from which BKRF4 was excluded. In these cells, PML NBs were found at the periphery of these foci.

None of the 24 subnuclear proteins co-localized with Cajal bodies or nuclear speckles (Table 1). The remaining seven subnuclear proteins (HSV US1, US1.5, UL26.5, UL54 and CMV UL30, UL69 and UL137) did not co-localize with any of the host NBs that we examined, and the size and shape of these structures tended to vary from cell to cell, depending on protein expression levels.

### Disruption of Cajal bodies

**Figure 5:**
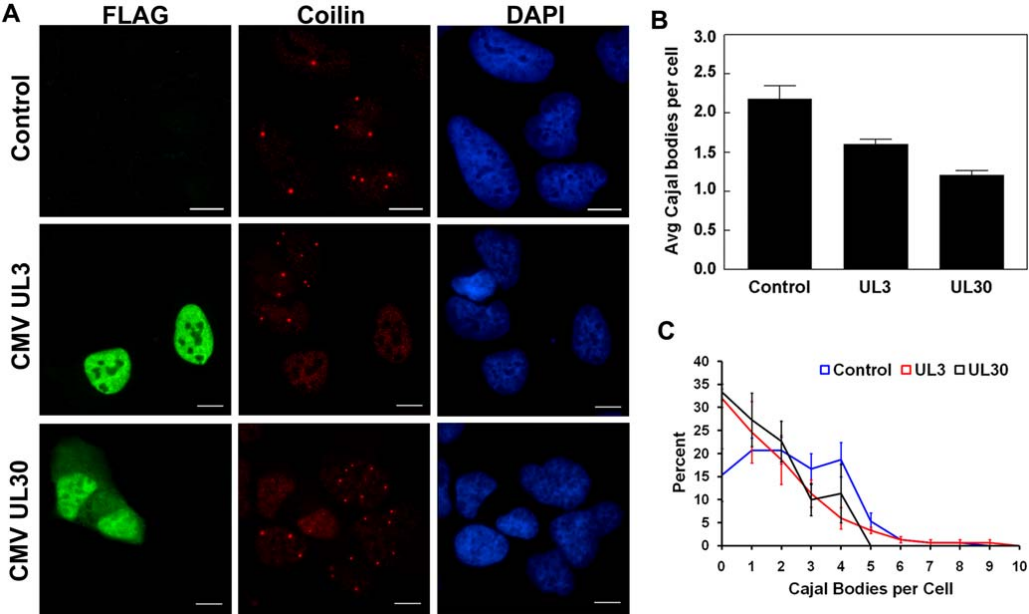
Disruption of Cajal bodies by CMV UL3 and UL30. (A) UL3- and UL30- expressing U2OS cells and untransfected control cells were stained with FLAG antibody to detect the viral protein (green) and coilin antibody to detect Cajal bodies (red) and counterstained with DAPI (blue). Scale bar  =  10 µm. (B) Histogram showing the average number of Cajal bodies per cell in cells prepared as in (A). Mean values±SE from 5 separate experiments are shown. (C) Data from samples in (B) were plotted to show the percentage of cells with a given number of Cajal bodies per cell. Mean values±SE from 3 separate experiments are shown.

Although none of the 24 subnuclear proteins analyzed co-localized with coilin at Cajal bodies in U2OS cells, we noticed a tendency for cells expressing CMV UL3 or CMV UL30 to have fewer or no coilin-positive bodies (Figure 5A). It should be noted that, although both UL3 and UL30 can localize to sub-nuclear bodies (Figure 2), in most of the cells the staining was pan-nuclear as illustrated in Figure 5A. We saw no obvious correlation between the presence or absence of subnuclear staining and the disruption of Cajal bodies (data not shown). The observed decrease in Cajal bodies was quantified by counting the number of Cajal bodies observed by IF microscopy in U2OS cells expressing and not expressing the viral protein. Untransfected U2OS cells contained between 0 and 6 Cajal bodies per cell, with an average of 2.2 Cajal bodies per cell, whereas cells expressing UL3 and UL30 had significantly lower averages of 1.6 (*p*<0.05) and 1.2 (*p*<0.01) Cajal bodies, respectively (Figure 5B). The effect of these proteins on the number of Cajal bodies was even more apparent when the number of cells with each of 0 to 6 Cajal bodies per cell was plotted (Figure 5C). The expression of UL3 and UL30 resulted in a 2-fold increase in the percentage of cells with no Cajal bodies and a similar decrease in the percentage of cells with 4 to 6 Cajal bodies per cell. These results indicate that CMV UL3 and CMV UL30 can cause the disruption and/or loss of Cajal bodies.

### Reorganization and disruption of PML bodies

We were particularly interested in examining the effects of viral nuclear proteins on PML NBs, since some viral proteins are known to stimulate lytic viral replication by disrupting PML NBs. As mentioned above, five proteins at least partially co-localized with PML NBs and three of these also noticeably altered the PML bodies in various ways (Figure 4A). CMV US32 and UL3 expression resulted in PML NBs with altered size and shapes relative to PML bodies in untransfected or vector-transfected cells (compare to control panel in Figure 4B). In addition, in cells in which CMV UL35 formed ring-shaped structures, PML was reorganized into semi-circular structures corresponding to the periphery of the viral protein donuts.

A small number of herpesvirus proteins are known to contribute to lytic infection by decreasing the number of host PML NBs but only a minority of the viral proteins have been tested for this phenomenon. Given the important roles of PML NB in antiviral defenses, apoptosis and other processes, we expanded our analysis of PML alterations beyond the 24 subnuclear proteins to include all 93 proteins with nuclear localization. The effect of these proteins on the size, shape and number of PML NBs was examined in U2OS cells (Table S1). Sixty-one of the 93 nuclear proteins had no apparent effect on PML NBs and only the three proteins mentioned above appeared to reorganize the PML NBs. These results indicate that most viral proteins with nuclear localization do not alter PML NBs even at relatively high expression levels.

**Figure 6:**
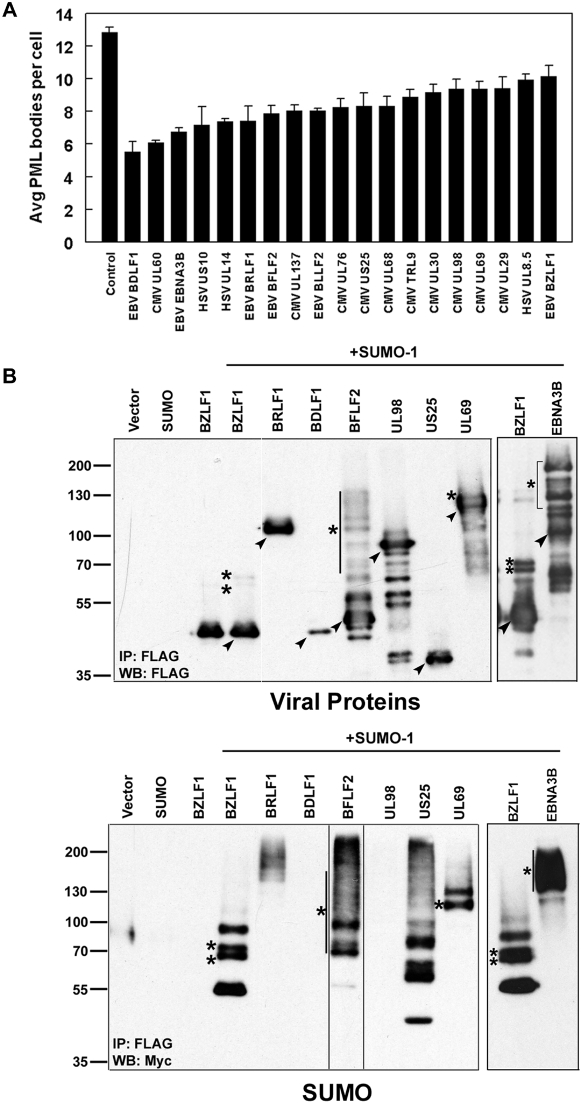
Quantification of PML disruption and detection of SUMOylated viral proteins that cause PML disruption. (A) Histogram showing the average number of PML bodies per transfected U2OS cell for each of the indicated viral proteins. Data is presented at the mean±SE (n = 3–8). (B) U2OS cells were co-transfected with the indicated viral protein and myc-tagged SUMO-1 as indicated, then subjected to anti-FLAG immunoprecipitation (IP) and Western blotting (WB) for FLAG (top panel) or SUMO (bottom panel). The SUMO-blot lane for BFLF2 is from the same blot, exposed for 1/3 the time of the remaining lanes in order to resolve individual SUMOylated protein forms. Arrowheads indicate the predicted molecular weight of the unmodified viral protein. Bands marked with an asterisk (*) represent higher molecular weight forms of the viral protein that were evident in the viral protein immunoblot and shown to contain SUMO in the SUMO-blot.

For any viral protein that was perceived to decrease or alter PML NBs (35 proteins), the number of PML NBs per cell was counted for 100 cells and compared to the same cells lacking viral protein expression. Although the number of PML NBs varies from cell to cell, untransfected U2OS cells contained an average of ∼13 PML bodies per cell (Figure 4B and Figure 6A). Of the 35 viral proteins for which PML NBs were counted, 16 decreased the number of PML NBs by 20% or less with most of these 16 proteins averaging between 11 and 14 PML bodies per cell. One of the viral proteins in our study was EBV BZLF1 (also called Zta or Zebra), which is known to disrupt PML bodies and was therefore used as a positive control [29]. At low levels of protein expression, BZLF1 had little effect on the number of PML bodies, however at higher expression levels PML bodies were severely disrupted (Figure 4B). On average, cells expressing various levels of BZLF1 had a modest yet significant (*p*<0.01) 21% decrease in the average number of PML NBs (Figure 6A). Eighteen viral proteins, from all three herpesvirus subfamilies, disrupted PML NBs to a greater degree than BZLF1, causing a 23% to 57% decreases in the average number of PML NBs per cell (Figure 6A) and examples of these observed effects are shown in Figure 4B. Qualitatively, some proteins appeared to require higher levels of protein expression before effects on PML were observed (CMV UL69, UL76, UL98 and EBV BZLF1 and BFLF2). For all of the19 proteins that significantly reduced the number of PML NBs, the size and shape of the PML NBs was not obviously affected. The sizes of all of the 19 PML-disrupting proteins as well as the PML-altering proteins in Figure 4A were verified by Western blotting (Figure S1).

PML body formation requires modification of PML by the small ubiquitin-like modifier, SUMO [26],[36], and SUMO-modified proteins such as BZLF1can disrupt PML NBs by competing with PML for limited cellular SUMO pools, resulting in inefficient SUMOylation of PML [29]. Therefore we analyzed the 19 proteins that disrupted PML NBs for potential SUMOylation sites using SUMOplot (www.abgent.com.cn/doc/sumoplot/). This identified 8 proteins (EBV BZLF1, BRLF1, BDLF1, BFLF2 and EBNA3B and CMV UL69, UL98 and US25) with at least one, high-probability potential SUMO modification site (Table S3). We tested whether these proteins were SUMOylated by co-expressing them with myc-tagged SUMO-1 in U2OS cells then immunoprecipitating the viral protein (Figure 6B). Consistent with previous reports, BZLF1 and BRLF1 were SUMO-1 modified and produced multiple higher molecular weight, SUMOylated forms (Figure 6B; also see Figure S1 for protein migration in the absence of exogenous SUMO). In addition, four other proteins (EBNA3B, BFLF2, UL69 and US25) also showed various higher molecular weight bands that reacted with the anti-myc antibody for SUMO-1, indicating that they too are SUMO-1 modified. No SUMO-1 bands were evident for BDLF1 and CMV UL98 (Figure 6B, bottom panel), although the low expression level of BDLF1 might have hampered the detection of SUMO-modified forms.

## Discussion

Herpesviruses are large DNA viruses with complex host interactions but only a minority of the encoded proteins have been functionally characterized. In order to gain insight into the possible functions of the many uncharacterized herpesviral proteins, we generated and screened genomic expression libraries from HSV, EBV and CMV for subcellular localization, resulting in the subcellular classification of 234 proteins. With only a few exceptions, previously reported protein localizations were confirmed, while new localization data was generated for approximately 160 previously unlocalized proteins, most of which originate from CMV. A genome-wide subcellular localization study similar to ours was recently published for the human gamma-herpesvirus HHV-8, which was found to have 51% cytoplasmic and 22% nuclear proteins (with 27% in both compartments) [37]. A comparison to our results with the other human gamma-herpesvirus, EBV, suggests that EBV has a higher proportion of nuclear proteins than HHV-8, with 31% in nuclear and 31% in cytoplasmic compartments. We have focused our attention on nuclear proteins for all three herpes viruses in our study, including several proteins that localized to subnuclear structures, in order to gain a more comprehensive understanding of how herpesviruses alter host nuclear bodies to support viral replication.

Many viruses encode proteins that interact with and/or alter the nucleolus [38]. Traditionally described as the site of ribosome biogenesis, the nucleolus is emerging as a multifunctional and dynamic nuclear body that can contain hundreds of different cellular proteins and is involved in several signaling pathways, including cell cycle control [39],[40]. Therefore, it is not surprising that some herpesvirus proteins would alter nuclear process through interactions with the nucleolus. Prior to this study, five HSV proteins (ICP0, UL24, UL54, US11, RL1), one CMV protein (UL76) and one EBV protein (EBNA5) were reported to associate with the nucleolus, in some cases causing nucleolar reorganization or redistribution of nucleolar components [41]–[45]. While ICP0 and EBNA5 were not included in our screen, we also found that the remaining four proteins were associated with the nucleolus, although to varying degrees. Although HSV UL24 and CMV UL76 are related proteins, their localization was not identical in our study. UL76 was almost exclusively located at the nucleolus, whereas UL24 was observed in the nucleolus, the nucleoplasm and in subcytoplasmic structures. UL24 has been reported to disperse nucleolin from the nucleolus during HSV infection [41], but we did not observe alterations in EBP2 localization, suggesting that UL24 may affect specific nucleolar components or may require other HSV protein for nucleolar remodeling. The former possibility is consistent with previous observations that UL24 did not alter the nucleolar localization of fibrillarin [41]. In addition to the differences in localization, UL76, but not UL24, caused a significant decrease in the number of PML NBs per cell. These differences would suggest that despite sequence similarities, the functions of these two proteins may vary.

In addition to previously known nucleolar proteins, our screen identified eight more proteins with nucleolar association; HSV UL27.5 and CMV TRL-5, TRL-7, TRL-9, UL29, UL31, UL108 and US33. None of these proteins have been functionally characterized, although deletion of the UL29, UL31 or UL108 genes are known to moderately inhibit CMV infection [46]. Our results suggest that these three proteins, as well as the remaining nucleolar proteins may contribute to viral infectivity through alteration of nucleolar functions.

None of the viral proteins in our screen co-localized with nuclear speckles or Cajal bodies, however HSV US1 (more commonly known as ICP22) was found to form small nuclear foci that are juxtaposed and similar in number to nuclear speckles (Figure S4). This pattern of pairing of ICP22 with nuclear speckles has been previously reported [12]. In addition, the nuclear speckles appeared to be remodeled in the presence of ICP22, becoming more separated and punctate, rather than elongated and interconnected (Figure S4). Since nuclear speckles are linked to transcription and splicing, these observations may be connected to the reported effects of ICP22 on cellular and viral gene expression [47],[48]. EBV BMLF1 (also called Mta) has also been reported to be associated with nuclear speckles in transfected cells [14], however this was not observed in our study possibly due to the low expression level of this protein.

In the course of studying the subnuclear viral proteins, we found that two previously uncharacterized CMV proteins, UL3 and UL30, significantly decreased the number of Cajal bodies per cell. UL3 and UL30 have little sequence similarity and vary considerably in their contribution to CMV infection in fibroblasts; UL3 is classified a non-essential gene, while deletion of UL30 caused a severe defect in viral replication [46]. The Meq protein from the avian herpesvirus MDV has been reported to associate with Cajal bodies [7], however, to our knowledge, CMV UL3 and UL30 are the first human herpesvirus proteins reported to associate with or disrupt these structures. Although the functions of Cajal bodies are not completely clear, they are strongly associated with transcription and the maturation of ribonuclear particles and only form when these processes are active [49]. Therefore the disruption of Cajal bodies by UL3 and UL30 may reflect inhibition of transcription or RNP formation.

There may also be a relationship between Cajal and PML NBs in that these bodies have been reported to be juxtaposed and to associate through the PIASy protein [50] . Therefore it may not be a coincidence that we also found UL3 and UL30 to affect PML NBs, albeit in different ways. UL3 formed NBs in about 20–30% of the cells expressing this protein and, in these cells, PML NBs were irregularly shaped and always associated with UL3 NBs, suggesting remodeling of PML NBs by UL3. On the other hand, UL3-expressing cells with pan-nuclear staining were unaffected in the number or shape of PML NBs. In contrast, Cajal body disruption was observed for US3-expressing cells that had either pan-nuclear or NB localization, indicating that the mechanisms by which UL3 affects Cajal and PML NBs differ and that Cajal body disruption can occur in the absence of PML reorganization. Unlike UL3, UL30 significantly decreased the number of PML NBs per cell. UL30 formed NBs in 40–60% of expressing cells, but neither PML nor Cajal NB disruption by this protein was dependent on NB formation. These effects of UL30 may be part of its important role in viral replication [46]. While the two proteins that we found to disrupt Cajal bodies also affect PML NBs, the reverse is not true, as we identified several viral proteins that disrupted PML NBs but had no noticeable effect on Cajal bodies.

Four viral proteins in addition to UL3 were observed to localize with and/or alter the morphology of PML NBs to various degrees; namely CMV US32, UL35 and UL80a and EBV BKRF4. The most extensive co-localization with PML was seen for US32, an uncharacterized protein that is not essential for viral replication in fibroblasts [46]. UL80a functions in capsid assembly and is homologous to HSV UL26.5 and EBV BDRF1 [51]. UL80a foci were often juxtaposed to PML NBs although this was not observed with the UL80a homologues. EBV BKRF4 was found extensively through the nucleus except for multiple spherical regions from which it was excluded, and which had PML in the periphery. BKRF4 has not been functionally characterized but its localization in the tegument portion of the virion indicates that it could exert its effects immediately after infection [52].

For CMV UL35 we observed that a high proportion (∼50%) of both 293T and U2OS cells expressing this protein contained donut-shaped UL35 NBs of various sizes and that all of the visible PML was associated with the surface or periphery of these structures. The UL35 gene produces two co-linear proteins at different times during infection (75 kDa UL35 and 22 Kda UL35a), which act to regulate transcription from the major immediate early promoter [53]. Western blots of our UL35-transfected cells predominantly showed the larger form of the protein (data not shown), suggesting that UL35 is responsible for the PML remodeling in our studies. Both UL35 and UL35a can bind UL82, which is known to associate with PML NBs through an interaction with hDaxx [54],[55]. Previous reports have noted an association of UL35 with PML NBs in human foreskin fibroblasts, and found that this association is increased in the presence of UL82 [54]. Our results extend previous results to show that UL35 can also remodel PML NBs in some cell backgrounds. In our system co-expression with UL82 did not noticeably alter UL35 localization or PML effects (data not shown).

Our screen of 94 viral proteins with nuclear localization identified 19 viral proteins that significantly decreased the number of PML NBs in cells, including several that had not been previously recognized to do so. Many of these proteins have not been functionally characterized but their ability to disrupt PML NBs suggests that they may contribute to viral infection at least in part by altering host nuclear processes. For tegument proteins or those expressed early in infection, the disruption of PML NBs may directly contribute to viral infection. For proteins that are only present later in infection, the disruption of PML NBs may not be the contribution of the protein to infection, as PML NBs would already be disrupted through the action of other proteins. Nonetheless, the observed PML effects show that these proteins alter pathways that are linked to PML NBs.

Of the 3 HSV proteins we found to disrupt PML NBs (UL8.5, UL14, US10), the cellular effects of one (UL14) have been previously studied. Interestingly, UL14 is a tegument protein that has been shown to block apoptosis [56]. Since PML NBs are important for apoptosis, the PML disruption we observed for UL14 may account for its anti-apoptotic effects. Note that it is also well established that HSV ICP0 disrupts PML NBs through degradation of the PML protein [57],[58]. While ICP0 was not included in our initial screen, we did confirm that it disrupted PML NBs in transfected U2OS cells and had a more dramatic effect on PML NBs than any of the other viral proteins we tested (data not shown).

Previously one CMV protein, IE1, was reported to disrupt PML bodies [59]. While IE1 was not in our screen, we have identified 10 additional CMV proteins with similar effects on PML disruption, which vary in their degree of importance for infection [46]. One of the proteins that we found to significantly decrease PML NBs (UL137) is from the region of the viral genome that is deleted from the AD169 laboratory strain, and hence may contribute to infection in specific cell backgrounds such as endothelial cells which are infected by clinical CMV isolates but not by AD169 [60]. The fact that CMV produces so many proteins that target PML likely reflects the importance of PML disruption and PML-related pathways for the CMV replication and persistence.

The BZLF1 EBV protein has been previously reported to disrupt PML NBs thereby promoting lytic infection. We also found BZLF1 to disrupt PML NBs in our assay but identified 5 additional EBV proteins that had a greater effect on PML NBs than BZLF1 (BDLF1, BFLF2, BLLF2, BRLF1, EBNA3B). Four of these proteins had predicted sites of SUMOylation and three (BRLF1, BFLF2 and EBNA3B) were confirmed to be SUMOylated in the transfected U2OS cells. PML must be SUMO-modified to form NBs, and other SUMO-modified proteins such as BZLF1 are known to disrupt PML NBs at least in part by competing for limiting amounts of SUMO thereby inhibiting PML SUMOylation [17],[29]. Therefore it is likely that competition for SUMO is part of the mechanism by which BRLF1 and EBNA3B disrupt PML NBs. BRLF1 (also called Rta) has been previously reported to be SUMOylated at three sites, which contributes to its ability to activate lytic gene expression [61], however BRLF1 was not sufficient to disrupt PML NBs when expressed in HeLa cells [29]. Our results show that BRLF1 is sufficient to disrupt PML NBs in U2OS cells, indicating that this effect may vary in different cell backgrounds and suggesting that BRLF1 and BZLF1 might cooperate in promoting lytic activation through PML disruption.

The finding that EBNA3B disrupts PML NBs was unexpected as EBNA3B is a latency protein and PML disruption by viral proteins is generally associated with activation of lytic infection. In fact EBNA3B is only expressed during latency III or the growth program in B-cells, which occurs when B lymphocytes are immortalized with EBV *in vitro* and in the lymphomas that arise when patients are immunosuppressed [62],[63]. How EBNA3B contributes to latent infection has not been clearly determined, although it may be able to regulate viral and cellular transcription [64],[65]. Since PML NBs are important for apoptosis and negatively affect cancer development [66],[67], our results suggest that EBNA3B may contribute to cell immortalization and/or malignant transformation by EBV by disrupting PML NBs. PML disruption by EBNA3B might also be important for counteracting antiviral responses to latent EBV infection. For example, the EBV EBER RNA molecules expressed in EBV latency have been shown to induce the interferon response, which activates expression of the PML protein [68],[69]. However, the number and size of PML NBs was found to be similar in EBV-positive and EBV-negative B cell lines [70], suggesting that one or more latency proteins may counteract the accumulation of PML. EBNA3B is normally expressed with EBNA3C. Although EBNA3C did not express well in our assay, it has been previously shown to be modified by SUMO-3 (but not SUMO-1) and to disrupt PML NBs when overexpressed [71]. This study also reported detectable levels of SUMO-3 modification of EBNA3B. However, this appeared to be less efficient than the extensive modification by SUMO-1 that we have observed, where EBNA3B was modified at least as efficiently as the BZLF1 positive control. The similarity in PML disruption by EBNA3B and EBNA3C suggests that they might act either redundantly or cooperatively in this role during latency III infection. It is striking that the functions of several EBV latent and lytic gene products are regulated by SUMOylation and it seems likely that this will also hold true for EBNA3B.

In addition to the SUMOylated EBV proteins, two CMV proteins that we identified as disrupting PML NBs, UL69 and US25, were shown for the first time to be SUMO-modified. While US25 has not been functionally characterized, UL69 is a partial homologue to HSV ICP27 (UL54) and EBV BMLF1 (Table S2) and is known to have multiple contributions to viral infection, including transcriptional activation, the export of viral RNA from the nucleus and induction of cell cycle arrest [72]–[74]. Our results suggest that UL69 may also influence viral infection by disruption of PML NBs and that one or more of the functions of UL69 may be regulated by SUMOylation. Neither ICP27 nor BMLF1 were found to disrupt PML NBs, likely reflecting their rather limited homology with UL69.

Our large-scale screening approach for viral protein localization has led to the subcellular characterization of 234 proteins, many of which have not been previously studied. These include 12 proteins that we showed to be nucleolar and 5 that localize to and/or remodel PML NBs. In addition we identified 18 proteins that reduce the number of PML NBs to a greater extent than EBV BZLF1, which was previously recognized for its ability to disrupt PML NBs. Strikingly, most of the proteins that localized with and/or disrupt host NBs are CMV proteins suggesting that this virus is particularly adept at manipulating host nuclear processes. Our studies have also shown that disruption of host PML NBs is not a general phenomenon of viral proteins even when expressed at high levels, but rather is a property of specific proteins. However, all three herpesviruses that we tested clearly have multiple mechanisms by which they can disrupt PML NBs, likely reflecting the importance of this phenomenon and of PML-related pathways for successful infection. PML NBs have been increasingly tied to key cellular processes including host defenses, apoptosis, senescence and DNA repair [17] , and our results provide a starting point for investigating how the identified viral proteins may affect these processes through PML disruption.

## Materials and Methods

### Expression constructs

Expression libraries for HSV-1 (strain 17), CMV (strain AD169) and EBV (strain B95-8) were generated by PCR amplification of each predicted open reading frame (ORF), according to the NCBI entries for each virus, from viral genomic DNA samples generated as described in [75]. Nineteen additional CMV ORFs (UL133-151) not present in AD169 were obtained by PCR amplification from the large Towne strain of CMV. In addition cDNAs for three spliced EBV ORFs were obtained from Dr. Elliott Kieff (EBNA3B and EBNA3C) and Dr. George Miller (BZLF1). cDNAs were inserted into the multicloning site in pMZS3F [32], with the aid of cloning robots, such that proteins are expressed fused to a C-terminal sequential purification affinity (SPA) tag comprised of a calmodulin binding peptide and a triple FLAG epitope.

### Immunofluorescence microscopy

Human 293T and U2OS cells were seeded into 6-well clusterplates on glass coverslips (700,000 cells/well) and transfected with expression plasmids using Lipofectamine 2000 (Invitrogen) as per the manufacturer's instructions, using a DNA to Lipofectamine 2000 ratio of 2 µg∶2 µl for 293T cells and 2 µg∶4 µl for U2OS cells. Transfected cells were fixed 40 h post transfection with 3.7% formaldehyde in PBS (20 min), permeabilized with 0.5% Triton X-100 in PBS (10 min), and blocked with 4% BSA in PBS (20 min) prior to incubation with primary and secondary antibodies in 4% BSA in PBS. Herpesvirus proteins were detected using either mouse anti-FLAG M2 (Sigma) or rabbit anti-FLAG (AbCam) antibodies. Nucleoli, PML bodies, nuclear speckles, Cajal bodies and ER were visualized using rabbit anti-EBP2 , rabbit anti-pan-PML (Chemicon), mouse anti-SC35 (AbCam), rabbit anti-coilin (Santa Cruz) and mouse anti-PDI (AbCam) antibodies, respectively. Primary antibodies were detected using either goat anti-mouse Alexafluor 488 or goat anti-rabbit Alexafluor 555 secondary antibodies (Molecular Probes). Coverslips were mounted onto slides using ProLong Gold antifade fluorescent mounting medium (Invitrogen) containing DAPI for visualization of nuclear DNA. Images were acquired using the 63× oil objective (NA 1.4) on a Leica DM IRE2 inverted fluorescent microscope. Images were processed using OpenLAB (ver.4.0.2) and Adobe Photoshop version 6.0 using only linear adjustments.

### Quantification of nuclear bodies

Quantification of Cajal and PML bodies was conducted in U2OS cells prepared as described above for fluorescence microscopy. For each herpesvirus protein, the number of nuclear bodies in each of 50 transfected cells was recorded. This data was used to calculate the average number of PML or Cajal bodies per transfected cell (histograms) and to quantify the frequency of a given number of nuclear bodies per cell within a population (line graphs). Statistical analyses (ANOVA and t-test) were conducted using GraphPad Prism (version 4.03) software.

### SUMOylation assay

U2OS cells in 10 cm plates were co-transfected with 6 µg each of pMZS3F expressing a viral protein and pCMVmycSUMO1 expressing myc-tagged SUMO-1 (M. MacPherson and P. Sadowski, in preparation). At 40 h post transfection, cells were washed twice with PBS and harvested. Cells were lysed with 200 µl lysis buffer (2% SDS, 10% glycerol, 62.5 mM Tris pH 6.8) plus 1 nM N-ethyl-maleimide (Sigma) and protease inhibitor cocktail (P8340, Sigma). Samples were boiled for 5 min, sonicated and clarified by centrifugation at 13,000× g for 15 min at 4°C. The viral protein was immunoprecipitated by diluting the samples to 1 ml with IP buffer (50 mM Tris pH 8.0, 150 mM NaCl, 1% NP-40) and incubating with anti-FLAG resin (Sigma) for 4 hr at 4°C, with shaking. Beads were washed three times for 30 min in IP buffer and protein eluted with 50 µl of 2× protein sample buffer (5% SDS, 20 mM Tris pH 8, 10% DTT, 20% glycerol). Samples were subjected to SDS-PAGE, transferred to nitrocellullose membranes and probed for myc using mouse anti-myc (1∶4000 dilution, Abcam) or FLAG using mouse anti-FLAG M2 (1∶20,000 dilution, Sigma). 9/10 of each sample was used for the anti-myc blot to detect SUMO and 1/10 of each sample was used for the anti-FLAG blot to detect total protein. Bands were detected with goat anti-mouse HRP (1∶5000) and Western Lighting chemiluminescent reagent (PerkinElmer).

## Supporting Information

Table S1 Subcellular Localization and PML Disruption Results for all Herpesvirus Proteins
(0.15 MB XLS)

Table S2 293T Cell Localizations of Proteins Conserved in HSV, CMV and EBV
(0.04 MB DOC)

Table S3 Potential SUMOylation sites in PML-disrupting Proteins
(0.03 MB DOC)

Figure S1 Size of Herpesvirus Proteins that Alter or Disrupt PML NBs. Extracts of cell expressing the indicated viral protein were analyzed by Western blotting using anti-FLAG antibody (A–C). The predicted molecular weights (MW) for the untagged proteins are noted below each lane and are not adjusted for the 10 kDa SPA tag. Positions of molecular weight markers are indicated. Separations between lanes on the gels with the same MW markers in A and B indicate different exposure times for each lane from the same gel, to optimize detection of the indicated protein. Both short and long exposures were included for the proteins in (C) to show the major bands in the short exposure and reveal SUMO-modified forms in the longer exposure. UL69 in (C) was detected on a separate gel. BKRF4, BZLF1, BRLF1 and UL69 have been previously reported to migrate anomalously slowly on SDS-PAGE. The majority of proteins that we observed to migrate slower than their predicted molecular weight are either highly basic (BLLF2, UL68, TRL9, UL137 and US25) or highly acidic (BKRF4, BZLF1, UL14, UL98), consistent with the known property of highly charged proteins to migrate slower than their molecular weight. Note that variability in protein expression levels seen in the Western blots is at least partially due to differences in the percentage of cells expressing each protein (caused by differences in transfection efficiency of the various plasmids).
(4.54 MB TIF)

Figure S2 Herpesvirus Proteins with Subcytoplasmic Localizations. 293T cells transfected with the indicated viral proteins were formaldehyde-fixed and stained with FLAG antibody to visualize the viral protein. Scale bar  =  10 µm.
(8.52 MB PNG)

Figure S3 Confirmation of ER Localization. 293T cells were transfected with the indicated viral proteins with subcytoplasmic localizations. Cells were fixed and stained with FLAG (red) to visualize the viral protein and protein disulphide isomerase (PDI, green) to identify the endoplasmic reticulum. Co-localization is indicated by yellow in the merged images. CMV US7 is known to be ER-associated while the localization of BDLF2 is previously unreported. Scale bar  =  10 µm.
(3.29 MB TIF)

Figure S4 US1 Alters Nuclear Speckles. Untransfected U2OS cells (A) and cells transfected with HSV US1 or US1.5 were fixed and stained for FLAG (red) and SC35 (green) to identify viral proteins and nuclear speckles respectively. Scale bar  =  10 µm.
(2.06 MB PNG)
