# Supplementary material for: Genome-Wide Screen of Three Herpesviruses for Protein Subcellular Localization and Alteration of PML Nuclear Bodies
Source: PLoS Pathog. 2008 Jul 11;4(7):e1000100. doi: 10.1371/journal.ppat.1000100 (PMC2438612; doi:10.1371/journal.ppat.1000100)
Supplement: Table S2 — 293T Cell Localizations of Proteins Conserved in HSV, CMV and EBV (0.04 MB DOC) [file ppat.1000100.s002.doc]

**Table S2: 293T Cell Localizations of Proteins Conserved in HSV, CMV and EBV**

| **VIDA Family #* and**  **Conserved Function** | **Virus** | **Protein** | **Localization** |
| --- | --- | --- | --- |
| 12  Unknown | HSV | UL7 | Pan-cellular |
|  | CMV | UL103 | Cytoplasmic |
|  | EBV | BBRF2 | Pan-cellular |
| 19  Transactivator/  RNA export | HSV | UL54 | Pan-nuclear |
|  | CMV | UL69 | Pan-nuclear |
|  | EBV | BMLF1 | Pan-nuclear |
| 21  Virion Assembly | HSV | UL25 | Pan-cellular, some cytoplasmic bodies |
|  | CMV | UL77 | Pan-cellular, some cytoplasmic bodies |
|  | EBV | BVRF1 | Pan-cellular |
| 23  Unknown | HSV | UL31 | Pan-cellular |
|  | CMV | UL53 | Pan-nuclear |
|  | EBV | BFLF2 | Pan-nuclear |
| 24  Capsid | HSV | UL18 | Pan-cellular |
|  | CMV | UL85 | Pan-cellular |
|  | EBV | BDLF1 | Pan-cellular |
| 27  Capsid Protease | HSV | UL26 | Pan-nuclear |
|  | CMV | UL80 | Pan-nuclear |
|  | EBV | BVRF2 | Pan-nuclear |

* from http://www.biochem.ucl.ac.uk/bsm/virus_database/all_19.html
