# Supplementary material for: Genome-Wide Screen of Three Herpesviruses for Protein Subcellular Localization and Alteration of PML Nuclear Bodies
Source: PLoS Pathog. 2008 Jul 11;4(7):e1000100. doi: 10.1371/journal.ppat.1000100 (PMC2438612; doi:10.1371/journal.ppat.1000100)
Supplement: Table S3 — Potential SUMOylation sites in PML-disrupting Proteins (0.03 MB DOC) [file ppat.1000100.s003.doc]

**Table S3**: Potential SUMOylation sites in PML-disrupting proteins.

| **Protein** | **SUMOplot results*** |
| --- | --- |
| EBV BZLF1 | LKGE (Lys-192) |
| EBV BRLF1 | LKPG (Lys-316) |
| EBV BDLF1 | VKLE (Lys-104) |
| EBV BFLF2 | VKAG (Lys-193) |
| EBV EBNA3B | VKKG (Lys-781)  AKIE (Lys-873)  AKQE (Lys-913) |
| CMV US25 | VKSP (Lys-136) |
| CMV UL69 | VKSP (Lys-136) |
| CMV UL98 | IKHE (Lys-214)  LKQG (Lys-261)  LKPP (Lys-352) |

*The amino acid sequences of 18 nuclear herpesvirus proteins that decreased the number of PML bodies in transfected U2OS cells were analyzed for potential SUMO-modification sites using the SUMOplot online resource (www.abgent.com/tool/sumoplot). The putative SUMOylation sites noted for the eight proteins in the table above were listed as having a high probability for SUMOylation (>0.70).
