# Supplementary figures and images for: Genome-Wide Screen of Three Herpesviruses for Protein Subcellular Localization and Alteration of PML Nuclear Bodies
Source: PLoS Pathog. 2008 Jul 11;4(7):e1000100. doi: 10.1371/journal.ppat.1000100 (PMC2438612; doi:10.1371/journal.ppat.1000100)

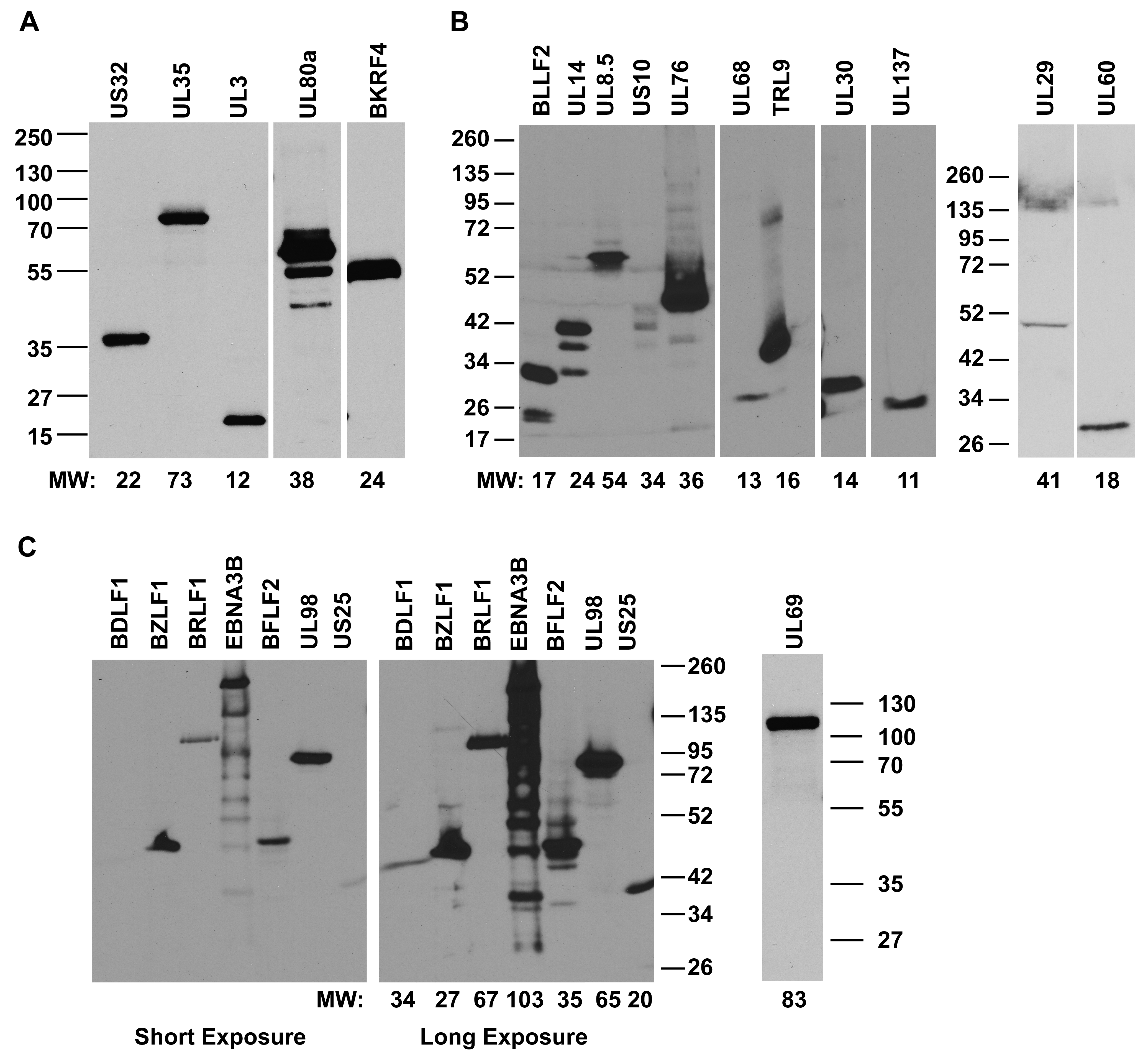

Supplement: Figure S1 — Size of Herpesvirus Proteins that Alter or Disrupt PML NBs. Extracts of cell expressing the indicated viral protein were analyzed by Western blotting using anti-FLAG antibody (A–C). The predicted molecular weights (MW) for the untagged proteins are noted below each lane and are not adjusted for the 10 kDa SPA tag. Positions of molecular weight markers are indicated. Separations between lanes on the gels with the same MW markers in A and B indicate different exposure times for each lane from the same gel, to optimize detection of the indicated protein. Both short and long exposures were included for the proteins in (C) to show the major bands in the short exposure and reveal SUMO-modified forms in the longer exposure. UL69 in (C) was detected on a separate gel. BKRF4, BZLF1, BRLF1 and UL69 have been previously reported to migrate anomalously slowly on SDS-PAGE. The majority of proteins that we observed to migrate slower than their predicted molecular weight are either highly basic (BLLF2, UL68, TRL9, UL137 and US25) or highly acidic (BKRF4, BZLF1, UL14, UL98), consistent with the known property of highly charged proteins to migrate slower than their molecular weight. Note that variability in protein expression levels seen in the Western blots is at least partially due to differences in the percentage of cells expressing each protein (caused by differences in transfection efficiency of the various plasmids). (4.54 MB TIF) [file ppat.1000100.s004.tif]

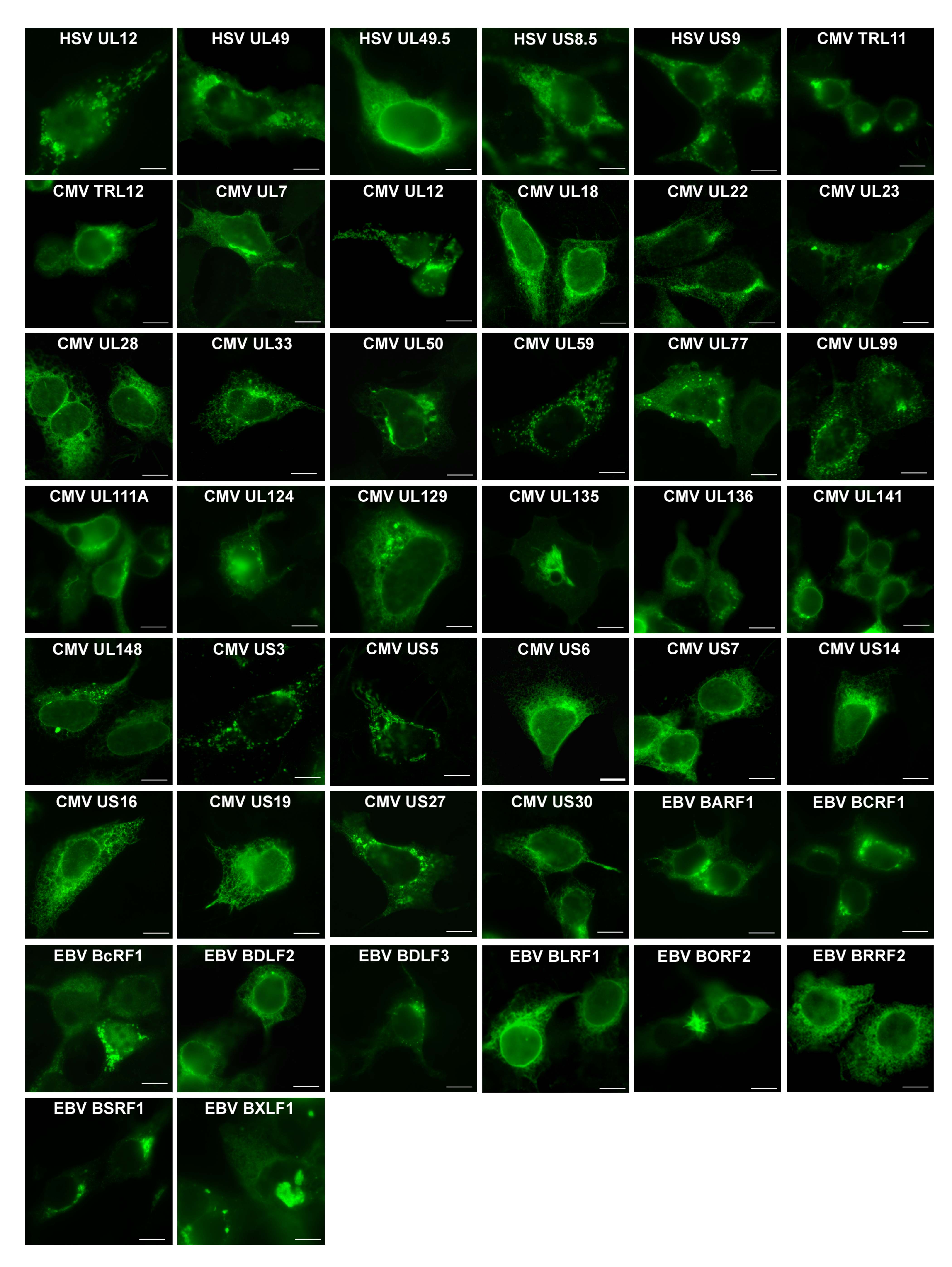

Supplement: Figure S2 — Herpesvirus Proteins with Subcytoplasmic Localizations. 293T cells transfected with the indicated viral proteins were formaldehyde-fixed and stained with FLAG antibody to visualize the viral protein. Scale bar = 10 µm. (8.52 MB PNG) [file ppat.1000100.s005.png]

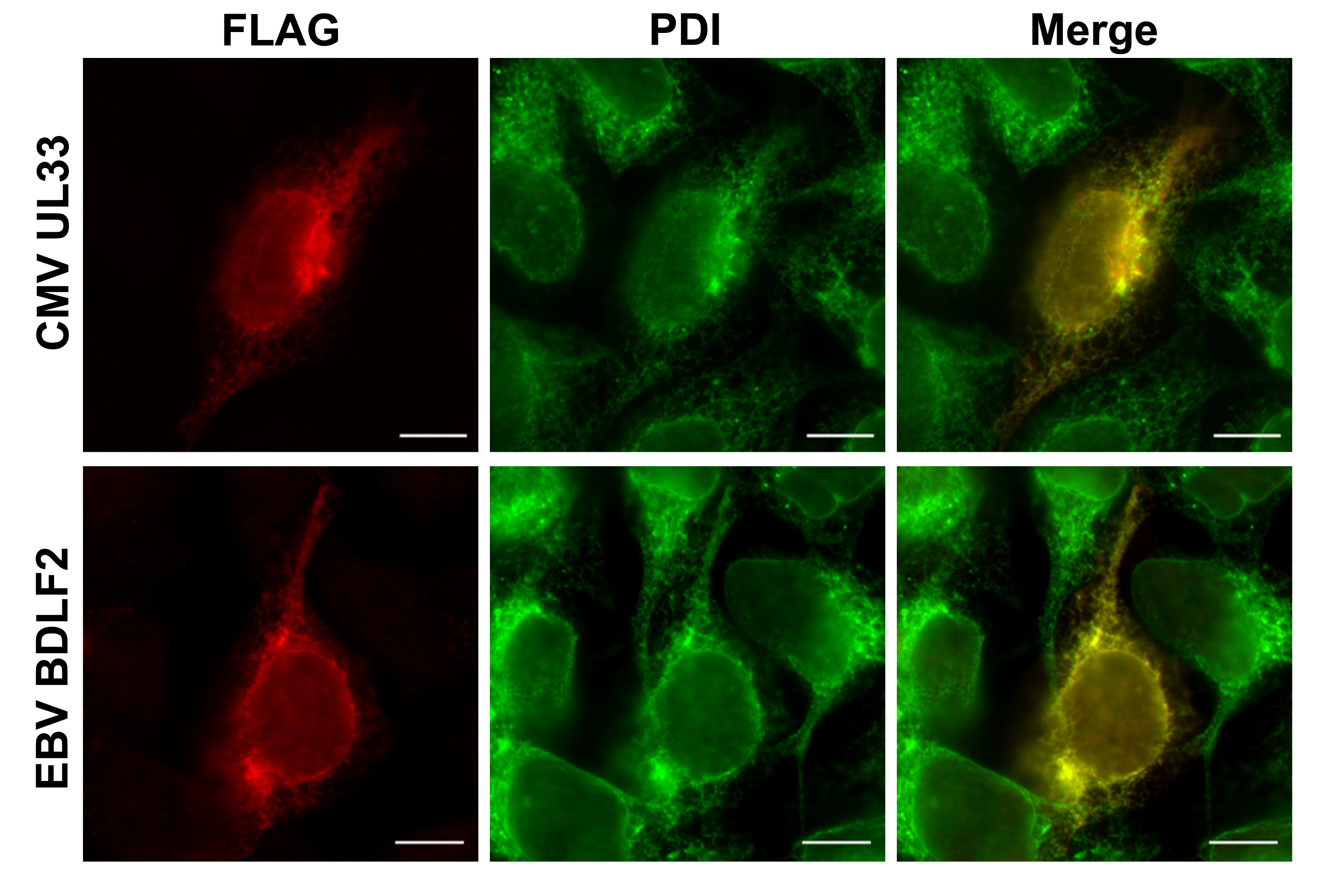

Supplement: Figure S3 — Confirmation of ER Localization. 293T cells were transfected with the indicated viral proteins with subcytoplasmic localizations. Cells were fixed and stained with FLAG (red) to visualize the viral protein and protein disulphide isomerase (PDI, green) to identify the endoplasmic reticulum. Co-localization is indicated by yellow in the merged images. CMV US7 is known to be ER-associated while the localization of BDLF2 is previously unreported. Scale bar = 10 µm. (3.29 MB TIF) [file ppat.1000100.s006.tif]

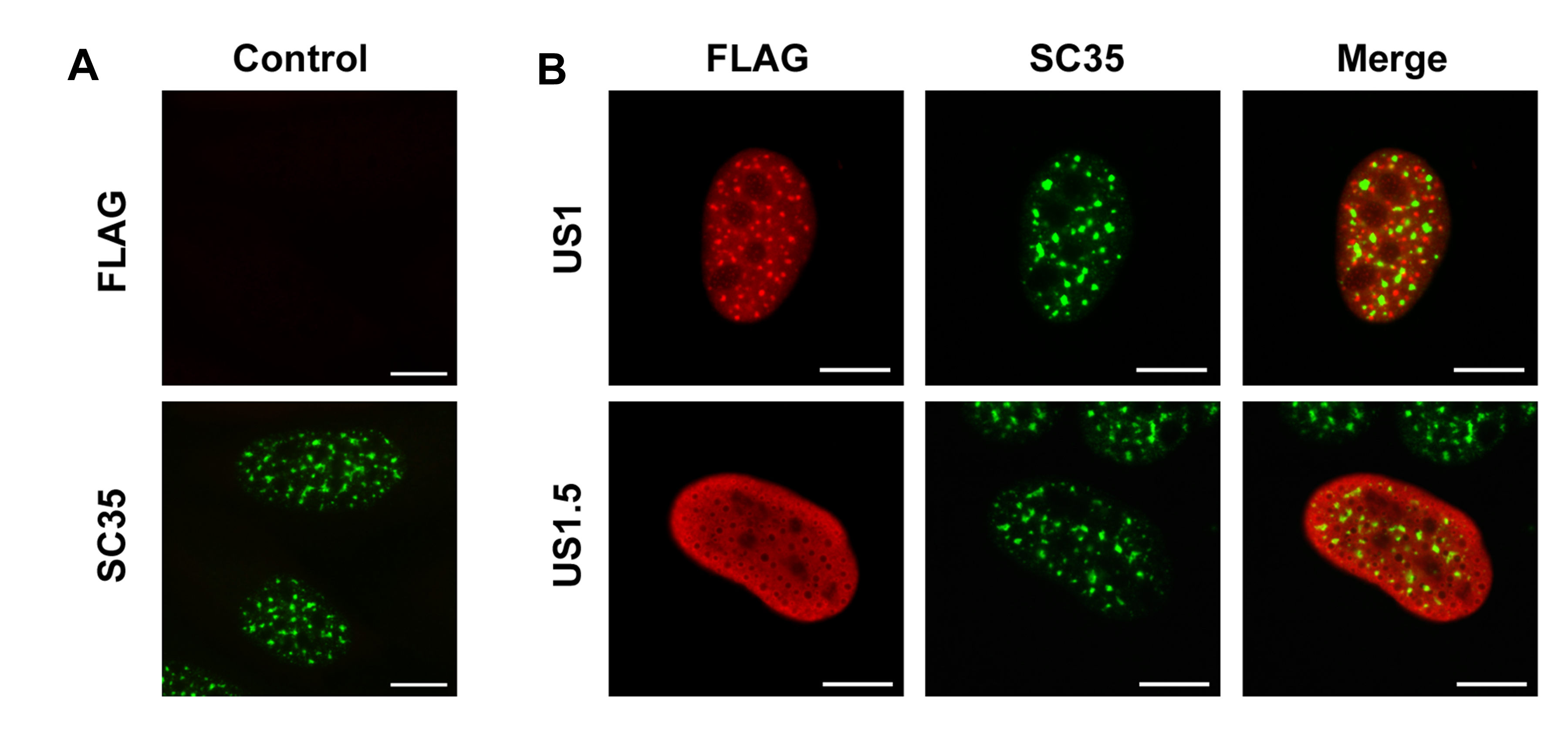

Supplement: Figure S4 — US1 Alters Nuclear Speckles. Untransfected U2OS cells (A) and cells transfected with HSV US1 or US1.5 were fixed and stained for FLAG (red) and SC35 (green) to identify viral proteins and nuclear speckles respectively. Scale bar = 10 µm. (2.06 MB PNG) [file ppat.1000100.s007.png]
